# Alkali-Etched NiCoAl-LDH with Improved Electrochemical Performance for Asymmetric Supercapacitors

**DOI:** 10.3390/nano13071192

**Published:** 2023-03-27

**Authors:** Liyin Hou, Xufeng Zhou, Lina Kong, Zhipeng Ma, Li Su, Zhaoping Liu, Guangjie Shao

**Affiliations:** 1 College of Environmental and Chemical Engineering, Yanshan University, Qinhuangdao 066004, China; 2 Key Laboratory of Graphene Technologies and Applications of Zhejiang Province and Advanced Li-Ion Battery Engineering Laboratory, Ningbo Institute of Materials Technology & Engineering, Chinese Academy of SciencesState, Zhejiang 315201, China; 3 State Key Laboratory of Metastable Materials Science and Technology, Yanshan University, Qinhuangdao 066004, China

**Keywords:** LDH, supercapacitor, alkali etached, cation vacancy

## Abstract

Hydrotalcite, first found in natural ores, has important applications in supercapacitors. NiCoAl-LDH, as a hydrotalcite-like compound with good crystallinity, is commonly synthesized by a hydrothermal method. Al${}^{3+}$ plays an important role in the crystallization of hydrotalcite and can provide stable trivalent cations, which is conducive to the formation of hydrotalcite. However, aluminum and its hydroxides are unstable in a strong alkaline electrolyte; therefore, a secondary alkali treatment is proposed in this work to produce cation vacancies. The hydrophilicity of the NiCoAl-OH surface with cation vacancy has been greatly improved, which is conducive to the wetting and infiltration of electrolyte in water-based supercapacitors. At the same time, cation vacancies generate a large number of defects as active sites for energy storage. As a result, the specific capacity of the NiCoAl-OH electrode after 10,000 cycles can be maintained at 94.1%, which is much better than the NiCoAl-LDH material of 74%.

## 1. Introduction

Supercapacitors (SC) have advantages over batteries on the part of exceptional cyclic stability and rate capability, which is helpful to relieve the pressure of the depletion of fossil fuel resources and accords with the strategy of sustainable development [1,2,3,4,5,6,7]. Conventional supercapacitors can be generally classified into two types according to the mechanism of storing charge. One is a double-layer capacitor, in which ions in the electrolyte are adsorbed on the surface of the electrode material due to electrostatic forces. The other type is a pseudo-capacitor, in which the charge is stored by a fast reversible chemical redox reaction of the electrode material [8,9,10,11]. Carbon-based materials are widely used as electrode materials for double-layer capacitors [12]. Carbon materials have excellent rate performance and cycling stability, but their low specific capacity leads to low energy density. Regarding pseudo-capacitor materials, such as conducting polymers, transition metal oxides, and transition metal hydroxides, with highly specific capacity and fast charge/discharge capability, a wide range of concerns have been raised [13,14,15]. However, their low conductivity and poor stability hinder their applicability to pseudo-capacitor materials after long charging and discharging processes at high current densities [16,17]. Layered double metal hydroxide (LDHs) is an ideal electrode material for supercapacitors because its lamellar structure provides a large specific surface area that boosts the double-layer capacitive performance [6,18,19,20]. Meanwhile, the transition metal elements in the lamellae provide a large number of electrochemical active sites that enhance pseudocapacitive performance. LDHs are composed of two or more metal elements with a hydrotalcite crystal structure [21,22,23]. It is well known that the capacitance of LDHs is derived from rapid redox reactions at the electrochemical interface [24,25]. Due to the conversion between different crystal phases in the electrochemical reaction, the stability of monometallic hydroxides is a matter of concern [7,26]. Wang et al. proved that adding trivalent aluminum elements into the mixed metal hydroxide layer of LDHs can improve the crystallinity and hydrophilicity of the ternary LDHs, which is conducive to electrolyte accessibility and charge transfer [22]. For the hydrotalcite material, the aluminum element plays an important role in promoting the formation of crystals. During the electrochemical reaction, nickel hydroxide and cobalt hydroxide have large surface activity and can provide rapid redox reactions. The aluminum hydroxide in the hydrotalcite structure has no electrochemical activity under a voltage window of 0–0.55 V. Qiu et al. demonstrated that NiCoAl-layered double hydroxide nanoplates with nanowires heterostructure exhibited considerably good electrochemical performance [27]. Wang et al. described a method for fabricating cationic vacancies in NiFe-LDHs with alkali etching to have good electrocatalytic properties [28]. According to the reported articles, we speculate that alkaline etching pretreatment of NiCoAl-LDH is an effective method to improve its electrochemical performance. Herein, we propose a simple method to improve the electrochemical performance of NiCoAl-LDH (NCAL) by alkali treatment process to remove part of the instable aluminum compounds without destroying the LDH structure. After alkali treatment, the content of Ni${}^{3+}$ and Co${}^{3+}$ in NiCoAl-OH (NCAO) showed an increasing trend [29]. A highly specific capacity of 259.5 mAh g${}^{-1}$ at 1 A g${}^{-1}$ and 168.0 mAh g${}^{-1}$ at 10 A g${}^{-1}$ is delivered for NCAO with capacity retention of 64.8%. The cycle performance under the current intensity of 10 A g${}^{-1}$ was evaluated. In particular, after 10,000 times of charge and discharge, the specific capacitance of NCAO can still be maintained at 94.1%, which is much better than that of NCAL (74.0%). NCAO//AC devices have an energy density of 68.5 Wh kg${}^{-1}$ at a power density of 256.1 W kg${}^{-1}$. When the power density is increased to 2.6 kW kg${}^{-1}$, the energy density can still be as high as 51.5 Wh kg${}^{-1}$.

## 2. Materials and Methods

### 2.1. Preparation of NiCoAl-OH

First, 0.09 mmol nickel nitrate hexahydrate, 0.02 mmol cobalt nitrate hexahydrate, 0.01 mm aluminum nitrate nonahydrate compound, and 0.4 mmol urea were dissolved in 40 mL deionized solution. Secondly, the solution was stirred for 0.5 h. When it was fully dissolved, it was transferred to a pressure vessel with polytetrafluoroethylene as the inner layer. It was then placed in an environment of 140 degrees Celsius and heat-treated for 14 h. Under the protection of N${}_{2}$, the as-prepared NiCoAl-LDH (NCAL) was treated at 60 ${}^{\circ }$C in the 6 M NaOH solutions for 6 h, and the product was named NCAO. When the layered double hydroxides were treated in high concentration alkali, the crystal form easily changed. Hence, NCAL was also treated in 10 M NaOH solutions as a comparative experiment.

### 2.2. Structural and Morphological Characterization

X-ray diffraction is a very effective method in the qualitative study of materials. X-ray (XRD) experiments were carried out under 40 KV, 100 mA, and 5 °C, using CuK alpha (La = 0.15406 nm) as a light source, scanning with 5 °C, and XRD experiments under the condition of 2 theta = 5∼70 °C. The micromorphology and composition of the tested materials were observed by scanning electron microscopy (SEM), a matched energy spectrometer (EDS). The surface morphology of the electrode was studied by using Hitachi S4800 SEM. The microstructure of the test material is analyzed in detail by TEM. A JEM-2010 transmission electron microscope was used for analysis. According to the nitrogen absorption isotherm, the specific surface area and pore diameter of the multistage channel were obtained by the Brunauer–Emett–Teller (BET) method.

### 2.3. Electrochemical Measurements

Firstly, the slurry of active material, binder (PTFE), and conductive additive (acetylene black) was mixed in a mass ratio of 8:1:1 and added to 10 ml of anhydrous ethanol to make a homogeneous mixture. Next, the mixed slurry ethanol was evaporated until the slurry was viscous and coated on 1*1 cm nickel foam to prepare the electrode. The three-electrode structure and asymmetric two-electrode device were tested by using Shanghai Chenghua (CHI660E) or a Neware battery testing machine. Here, 6 M KOH aqueous solution was used as the electrolyte, a corresponding Hg/HgO electrode (6 M KOH) was used as the reference electrode, and a graphite electrode with a highly specific surface area was used as the counter electrode. The capacities of the supercapacitor were calculated by the following formula [30]:

(1) C=IΔt/m

where *C* is the discharge specific capacity (mAh g${}^{-1}$), *I* is the current density (A g${}^{-1}$) used for the test, and *m* is the mass of the active materials (g).

## 3. Results and Discussion

**Figure 1:**
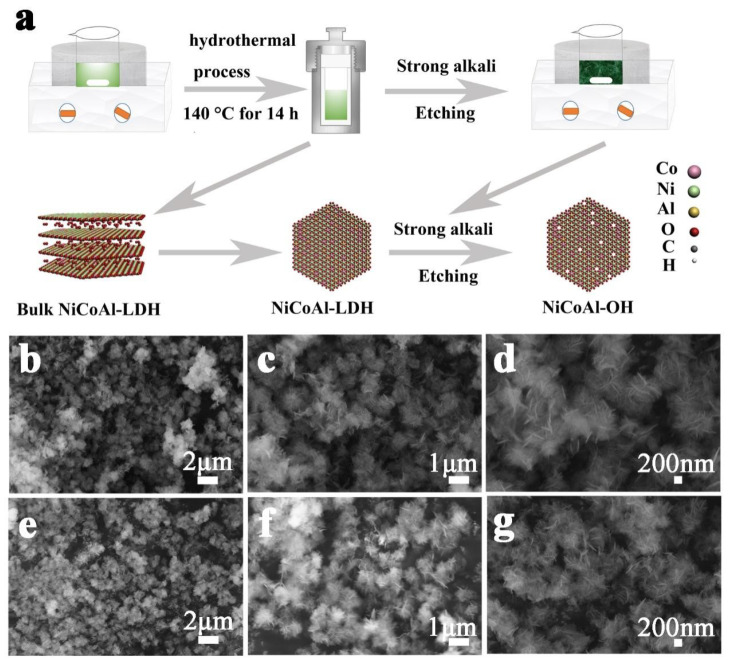
(**a**) The illustration of the preparation process and structures of NiCoAl-LDH and NiCoAl-OH. (**b**–**d**) SEM images of NiCoAl-LDH. (**e**–**g**) SEM images of NiCoAl-OH.

**Figure 2:**
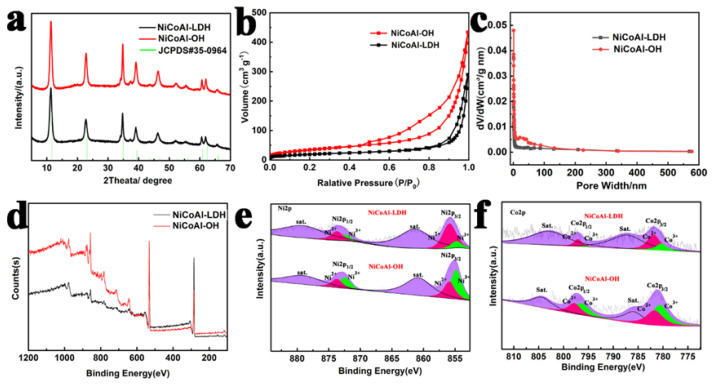
(**a**) Typical XRD patterns of the as-prepared NiCoAl-LDH and NiCoAl-OH. (**b**) N${}_{2}$ sorption isotherms of NiCoAl-LDH and NiCoAl-OH. (**c**) Pore size distribution of NiCoAl-LDH and NiCoAl-OH. (**d**) XPS spectra of NiCoAl-LDH and NiCoAl-OH. High-resolution XPS measurements of (**e**) Ni 2p${}_{3/2}$ and Ni 2p${}_{1/2}$, (**f**) Co 2p${}_{3/2}$ and Co 2p${}_{1/2}$.

A typical synthetic process of NCAO is shown in Figure 1a. First, LDHs were synthesized by the hydrothermal method. By treatment with a strong alkali of 6 M NaOH, the Al element on the surface of NCAL was removed, thereby forming cation vacancies. Moreover, these cation vacancies also affect the valence state of nearby metals, and the increase of Ni${}^{3+}$ and Co${}^{3+}$ contents compensates for the structural instability caused by the removal of Al${}^{3+}$ (Figure 2e,f). From the SEM photos of Figure 1, we can see that the morphology of NCAL has no obvious change under alkaline conditions, but it is still petal-shaped after the removal of Al. As can be seen in Figure 2a, the diffraction corresponds well to the (003), (006), (012), and (018) diffraction of a typical hydrotalcite-like structure (JCPDS: 35-0964) [4]. Under the alkaline condition, the phase state of the crystal does not change significantly, which shows a good microstructure retention [10,15]. It can also be clearly observed in Figure S1 that with a concentration of 10 M NaOH, the original petal-like morphology completely disappeared and smaller nanosheets were generated. We also found a significant change in the crystal form of NCAL when the concentration of NaOH solution was increased to 10 M, as shown in Figure S2. The original characteristic diffraction peaks of LDH at 10.9${}^{\circ }$, 22.1${}^{\circ }$, and 33.5${}^{\circ }$ disappeared, which may imply that the LDH is unstable in a high concentration alkali solution, and structural reorganization occurs. The XRD patterns are consistent with PDF#30-0443 and PDF#14-0117, suggesting that the original NCAL was transformed into the hybrid state of Co(OH)${}_{2}$ and Ni(OH)${}_{2}$.

Through the nitrogen absorption–desorption experiments of NCAL and NCAO samples, the pore and surface properties of hydrotalcite were obtained. The black curve and red curve with clear adsorption–desorption hysteresis loops show a representative IV isotherm (Figure 2b), manifesting that NCAL and NCAO has a mesoporous structure. Compared with the specific surface area of NCAL’s 66 m${}^{2}$ g${}^{-1}$, NCAO samples have a specific surface area of of 117 m${}^{2}$ g${}^{-1}$ and use Brunauer–Emett–Teller (BET) methods [31]. The corresponding pore size distribution of NCAO samples by the Brunauer–Emett–Teller (BJH) method is calculated. The pore-size distribution curve shows that the average pore size of NCAL is 34 nm, and the average pore size of NCAO is about 22 nm [31]. Its rich mesoporous structures pores can make the electrolyte penetrate into the deep layer and then improve its electrochemical properties. The chemical composition and surface electronic state of NCAL and NCAO were studied by the XPS technique. The binding energy state of the Ni, Co, Al, O, and C elements can be clearly seen from Figure 2d,e. The Ni 2p and Co 2p spectra for all samples can be deconvoluted into 2p3/2 and 2p1/2 due to the spin-orbit coupling, and each main peak is accompanied by a satellite peak [32]. For NCALs nanosheets, the peaks at 855.5 and 856.5 eV in the Ni 2p3/2 spectrum belong to Ni${}^{2+}$ and Ni${}^{3+}$ coordinated by OH${}^{-}$, respectively. The peak of 861.4 eV can be attributed to the satellite peak of Ni${}^{2+}$. Additionally, the peak of 863.1 eV can correspond to Ni${}^{3+}$ in NCAL, indicating the successful synthesis of NCAL. For both NCAL and NCAO nanosheets, the percentage content of Ni${}^{3+}$ increases significantly. It is found that Al defects will cause the transformation from Ni ${}^{2+}$ to Ni${}^{3+}$. Similarly, the content of Co${}^{3+}$ also increases. In addition, the smaller the particle size in the LDH, the more unsaturated coordination sites it has.

**Figure 3:**
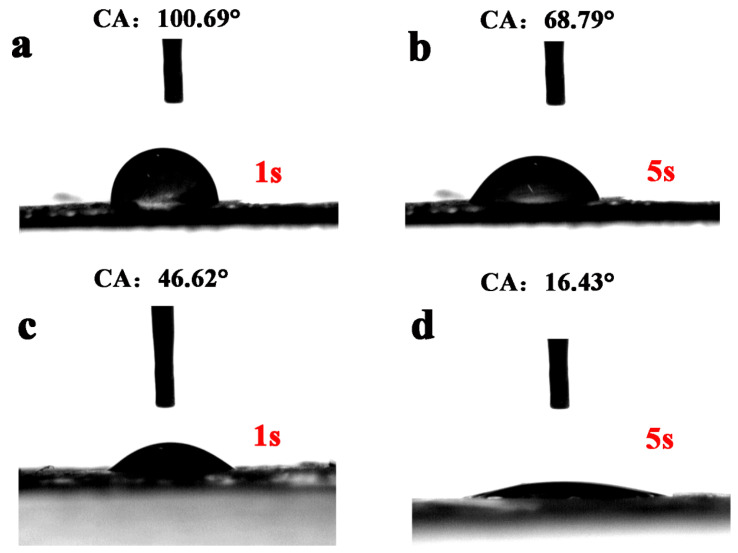
The contact angle of (**a**) NiCoAl-LDH at 1s, (**b**) NiCoAl-LDH at 5s, (**c**) NiCoAl-OH at 1s, and (**d**) NiCoAl-OH at 5s.

**Figure 4:**
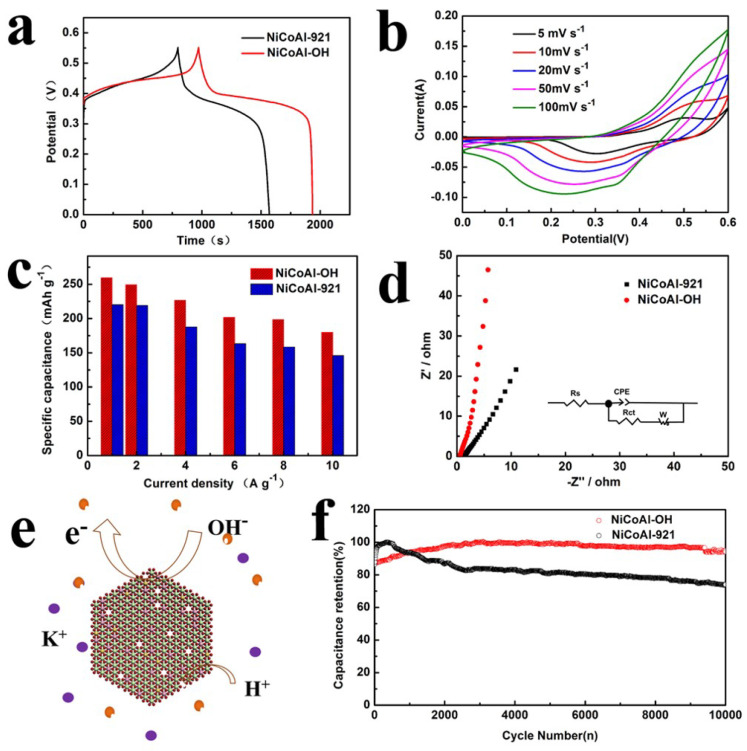
(**a**) Galvanostatic charge/discharge curves of the NiCoAl-LDH and NiCoAl-OH electrodes at a current density of 1 A g${}^{-1}$. (**b**) CV curves of the NiCoAl-OH electrodes at various scan rates of 5–100 mV s${}^{-1}$. (**c**) The specific capacity of the NiCoAl-LDH and NiCoAl-OH electrodes at different current densities. (**d**) Nyquist plots of NiCoAl-LDH and NiCoAl-OH electrodes. (**e**) Schematic illustration of the charge storage mechanism of NiCoAl-OH electrodes. (**f**) Cycling performance at 5 A g${}^{-1}$ of NiCoAl-LDH and NiCoAl-OH electrodes in 6 M KOH.

On the other hand, NCAL requires that its surface is rich in hydroxyl radical to make its hydrophilicity better. When drops of water fall on the NCAO, the water spreads immediately (as shown in Figure 3c,d). The alkali-etched NCAO has better hydrophilicity compared to NCAL. This result also confirms changes in the surface structure of the material surface. At the same time, the experiment also proved that the surface structure of the substance at its interface has changed. This result gives us a lot of inspiration. For LDH with an Al element, the surface treatment will be beneficial to improve the surface chemical reactivity. In the past, electrochemical reactions have relied on the mobility of OH${}^{-}$ at low current density because the OH${}^{-}$ ions are almost completely diffused to the electrode surface. The NCAO with good hydrophilicity helps the electrolyte transfer from the aqueous system to the surface of the active material quickly. At the same time, the aqueous solution of KOH has a larger contact area with the electrode surface, effectively providing a more active specific surface area. The electrochemical performance of the NCAO-electrode was tested by constant current charging and discharging [33]. NCAO samples and comparative samples were tested in a three-electrode cell using an Hg/HgO reference electrode and a 6M KOH aqueous electrolyte. Figure 4a show the GCD curve of NCAL and NCAO in the potential range of 0–0.55 V. The electrochemical characteristics of NCAO are analyzed in detail by using the cyclic voltammetry curve obtained at the scanning rate of 5∼100 mV s${}^{-1}$ (as shown in Figure 4b). When the scanning rate is increased, the kinetics will play out over the course of the entire capacity. The results show that the shift of the oxidation peak is mainly caused by the charge dispersion polarization on the surface of the electrode Similarly, the reduction peak shifts to lower voltages for the same reason. The NCAL electrodes have a capacity of 259.5 mAh g${}^{-1}$, 249.5 mAh g${}^{-1}$, 226.7 mAh g${}^{-1}$, 201.7 mAh g${}^{-1}$, 198.4 mAh g${}^{-1}$, and 168 mAh g${}^{-1}$ at current densities of 1, 2, 4, 6, 8, and 10 A g${}^{-1}$, respectively (Figure 4c). When the current density is increased from 1A g${}^{-1}$ to 10A g${}^{-1}$, the capacity retention rate is 65%. In comparison, NCAL, which has not been etched, has a lower capacity than that of NCAO. In comparison, NCAL, which has not been etched, has a lower capacity than that of NCAO. This may be due to a change in the material. At the same time, the increase of surface defects also increases the active sites of the electrochemical reaction. As shown in Figure 4e, the alkali-etched NCAO surface has a lot of cation vacancies, which is favorable for adsorbing OH${}^{-}$, and the hydrophilicity of the surface is favorable for the wetting of the electrolyte. This is consistent with the material wettability discussed above. The charging and discharging mechanism of NCAO is [15,34]:

$${\mathrm{Ni}(\mathrm{OH})}_{2}+{\mathrm{OH}}^{-}⇌\mathrm{NiOOH}+H_{2}O+e^{-}$$

$${\mathrm{Co}(\mathrm{OH})}_{2}+{\mathrm{OH}}^{-}⇌\mathrm{CoOOH}+H_{2}O+e^{-}$$

**Figure 5:**
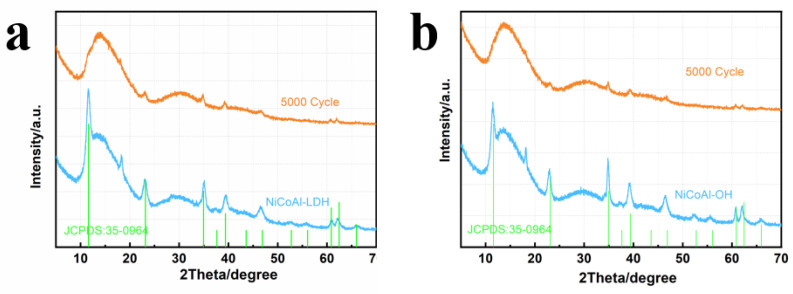
XRD patterns of electrodes of (**a**) NiCoAl-LDH before and after 5000 cycles, and (**b**) NiCoAl-OH before and after 5000 cycles.

Accordingly, the presence of cationic metal vacancies facilitates the adsorption of a large amount of OH- on the surface of the material, which is beneficial for the electrochemical reactions [28]. From the alkali etched NCAO material, it is found that the charge transport resistance of NCAO material decreases significantly by electrochemical impedance spectroscopy (EIS) test, and better results are obtained (as shown in Figure 4d) [17,35]. The Rs value did not change significantly in the high-frequency region. However, the Rct of NCAO is 0.3ω lower than that of ordinary of the NCOL (0.5ω), indicating that NCAO has lower internal resistance, thus improving its capacitance performance. The cyclicity of the NCAO electrode is further evaluated by the determination of the maintenance capacity at the ampere density of 10 Ag${}^{-1}$ (Figure 4f). The results show that after 10,000 cycles of charge and discharge, the specific capacity of the NCAO is still 94.1% of the initial value, which is obviously better than that of NCAL. In the plots, we also found that NCAL has an obvious activation process in the initial 500 cycles, and a process of capacity decay afterwards. This may be caused by the partial dissolution of Al${}^{3+}$ during rapid charge and discharge, resulting in a reduction in that leads to the decline of electrochemical performance. However, the long cycle curve of the alkali-etched NCAO shows a relatively stable trend, indicating that the pretreatment has a certain effect on the structural stability. Therefore, we measured the XRD patterns of NCAL and NCAO electrodes prepared under the same conditions before and after 5000 cycles. As can be seen in Figure 5a,b the diffraction peaks of the NCAL electrode after 5000 cycles shifts to smaller angles, indicating that the dissolution of Al and the insertion of more OH- leads to the increase of the lattice constant during charge and discharge. However, the structure of NCAO treated by alkali remains stable before and after 5000 cycles, and the XRD pattern does not change.

**Figure 6:**
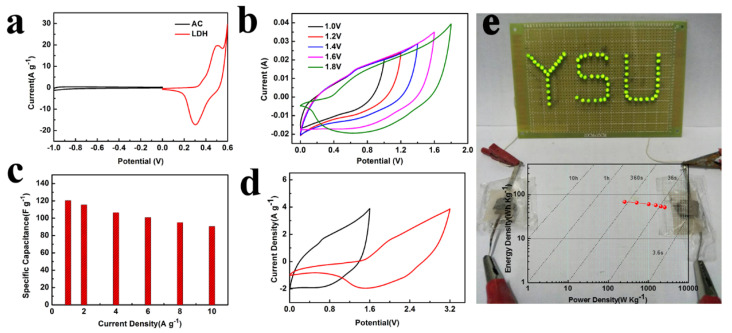
(**a**) CV curves of AC electrode and NiCoAl-OH electrode at a scan rate of 5 mV s${}^{-1}$. (**b**) CV curves of NiCoAl-OH//AC device under different voltage windows. (**c**) The specific capacity of the NiCoAl-OH//AC device at different current densities. (**d**) CV curves of a single hybrid device and two hybrid devices connected in series. The single device can sustain 1.6 V, such that the series connection of two capacitors can supply output potential up to 3.2 V. (**e**) Photographs of two hybrid devices connected in series, which can drive 64 green light-emitting diodes (LEDs) and Ragone plot of the NiCoAl-OH//AC device.

We assembled asymmetric supercapacitors and performed a series of electrochemical tests on them. Therefore, this project intends to use NCAO as the positive electrode and the AC as the counter electrode. First of all, under the fixed scanning rate of 10 mV s${}^{-1}$, the mass ratio of the two electrodes is calculated by using the integrated region of the CV curve of the positive and negative electrodes (as shown in Figure 6a). According to formula, the mass ratio of AC to NCAO is approximately 7.1 [30]:

(2) $$\frac{m_{+}}{m_{-}}=\frac{C_{-}\Delta V_{-}}{C_{+}\Delta V_{+}}$$

Here, m${}_{+}$ refers to the positive quality, m${}_{-}$ refers to the negative quality, C refers to the material specific capacity (mA hg${}^{-1}$), and ΔV refers to the voltage window. Figure 6b shows shows the CV curves of the asymmetric supercapacitors of each voltage window. Surprisingly, it was found that when the voltage window exceeded 1.6 V, there was a significant electrochemical polarization phenomenon. In this paper, we select the voltage range of 0–1.6 V and analyze it in detail. Figure 6c shows the capacities of the NCAO//AC device at various current densities. It can be seen that when the current density is 1 A g${}^{-1}$, the discharge capacity of the ASC device is 120.4 F g${}^{-1}$. The specific capacity of the electrode is decreased at 20 A g${}^{-1}$, the specific capacity of the electrode is still at the high value of 90.6 F g${}^{-1}$. Figure 6e shows the relationship between power density and energy density of NCAO//AC supercapacitors at various current densities. Because of its great superiority in power density, its energy density has also become an important index to evaluate the supercapacitor. According to the weight of the effective material, under the power density of 256.1 W kg${}^{-1}$, the energy density is 68.5 W h.kg${}^{-1}$. While increasing to 2.6 kW kg${}^{-1}$, its energy density still reaches 51.6 Wh kg${}^{-1}$. In this paper, the energy density and power density of related devices are compared and analyzed, as follows: the energy density of NiCo-LDHs-rGO// AC 47.1 Wh kg${}^{-1}$ at 399.9 W kg${}^{-1}$ [36], Ni-Co LDH-G//AC at 40.6 Wh kg${}^{-1}$ at 400 W kg${}^{-1}$ [37], NCNRs-NCNSs// AC at 22.8 Wh kg${}^{-1}$ at 374.5 W kg${}^{-1}$ [38], and NiCo${}_{2}$O${}_{4}$/NiCo-LDH// AC at 49 Wh kg${}^{-1}$ at 750 W kg${}^{-1}$ [39]. The NCAO//AC asymmetric supercapacitor with a unique structure was found to exhibit high energy density and power density. The voltage window of a single hybrid device is 1.6 V, and the charge and discharge voltage window of the two series devices can be increased to 3.2 V. In order to further explore the application of NCAO//AC device in real life, we connected two devices in series to illuminate the ’YSU’ plate composed of 64 small LED bulbs. It can be seen that the device has good capacity characteristics and will have great application prospects in the future.

## 4. Conclusions

In summary, the alkali-treated NCAO has a cation vacancy defect, and the content of Ni${}^{3+}$ and Co${}^{3+}$ increases, which changes the surface activity of the material. At the same time, a large amount of exposed OH- brings about good hydrophilicity of the material, which is beneficial to the infiltrating and adsorption of the electrolyte and boosting the electrochemical performance. A highly specific capacity of 259.46 mAh g${}^{-1}$ at 1 A g${}^{-1}$ and 168 mAh g${}^{-1}$ at 10 A g${}^{-1}$ with a specific capacity retention of 64.75% is delivered for NCAO. It is worth noting that the specific capacity of the NCAO electrode after 10,000 cycles can be maintained at 94%.

## Data Availability

The data that support the findings of this study are available from the corresponding author G.S. upon reasonable request.

## Supplementary Materials

The following supporting information can be downloaded at: https://www.mdpi.com/article/10.3390/nano13071192/s1, Figure S1: (a) SEM of NiCoAl-921, (b) TEM of NiCoAl-921, (c) SEM of 6M NaOH etched NiCoAl-LDH, (d) TEM of 6M NaOH etched NiCoAl-LDH, (e) SEM of 10M NaOH etched NiCoAl-LDH, (f) TEM of 10M NaOH etched NiCoAl-LDH. Figure S2: XRD pattern of alkali etaching NiCoAl-LDH with different concentration.
