# Supplementary material for: Alkali-Etched NiCoAl-LDH with Improved Electrochemical Performance for Asymmetric Supercapacitors
Source: Nanomaterials (Basel). 2023 Mar 27;13(7):1192. doi: 10.3390/nano13071192 (PMC10097225; doi:10.3390/nano13071192)
Supplement: Supplementary file 1 [file nanomaterials-13-01192-s001.zip › nanomaterials-2270684-supplementary.pdf]

nanomaterials

Supporting Information

Alkali-Etched NiCoAl-LDH with Improved Electrochemical Performance for Asymmetric Supercapacitors

Liyin Hou [1,2], Xufeng Zhou [2], Lina Kong [1], Zhipeng Ma [1], Li Su [1], Zhaoping Liu [2,*,†] and Guangjie Shao [1,3,†*]

[1] College of Environmental and Chemical Engineering, Yanshan University, Qinhuangdao 066004, China
[2] Key Laboratory of Graphene Technologies and Applications of Zhejiang Province and Advanced Li-Ion Battery Engineering Laboratory, Ningbo Institute of Materials Technology & Engineering, Chinese Academy of Sciences, Zhejiang 315201, China
[3] State Key Laboratory of Metastable Materials Science and Technology, Yanshan University, Qinhuangdao 066004, China
* Correspondence: liuzp@nimte.ac.cn (Z.L.); shaoguangjie@ysu.edu.cn (G.S.)
† These authors contributed equally to this work.

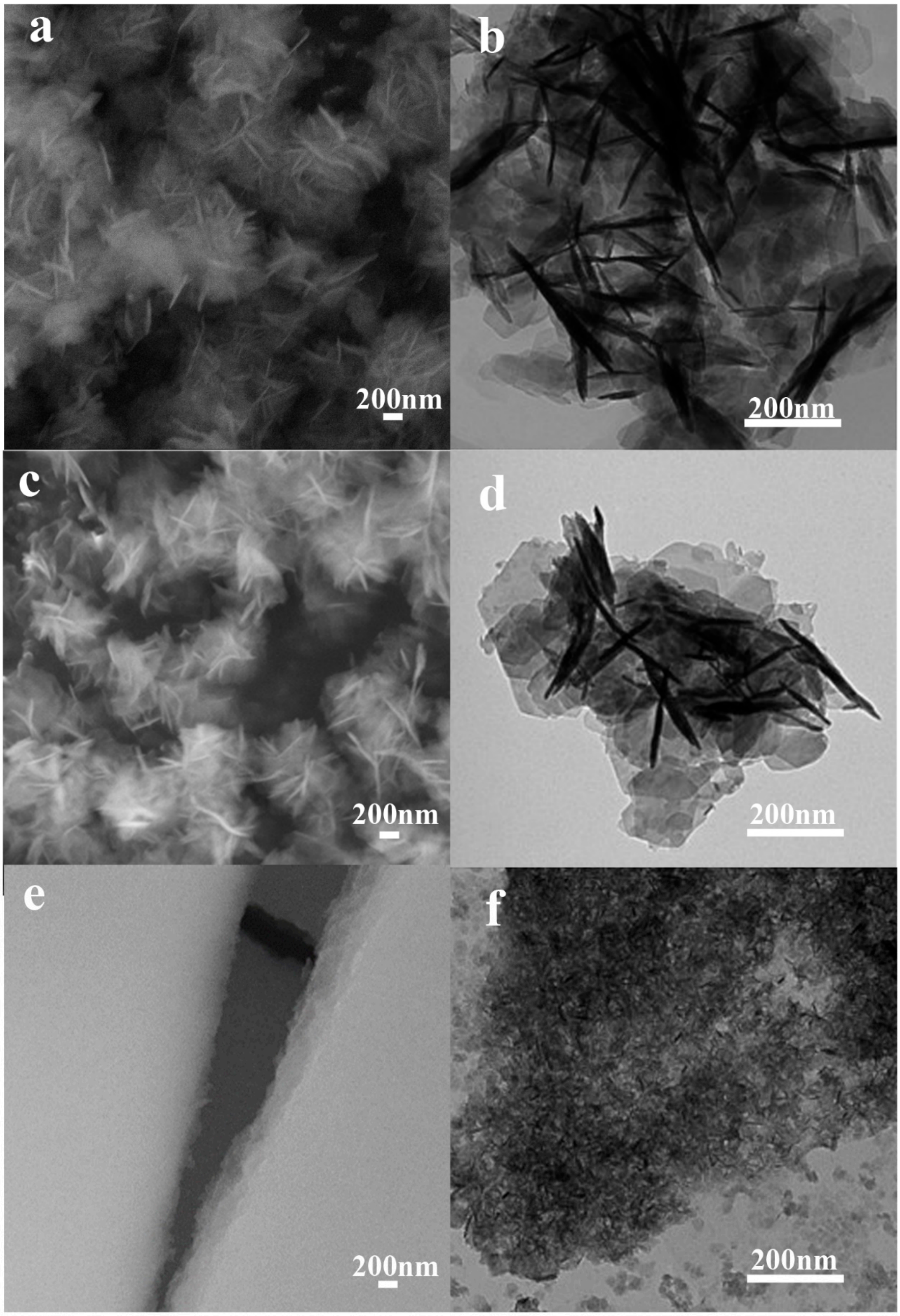

Figure S1. (**a**) SEM of NiCoAl-921, (**b**) TEM of NiCoAl-921; (**c**) SEM of 6M NaOH etched NiCoAl-LDH, (**d**) TEM of 6M NaOH etched NiCoAl-LDH; (**e**) SEM of 10 M NaOH etched NiCoAl-LDH, (**f**) TEM of 10 M NaOH etched NiCoAl-LDH.

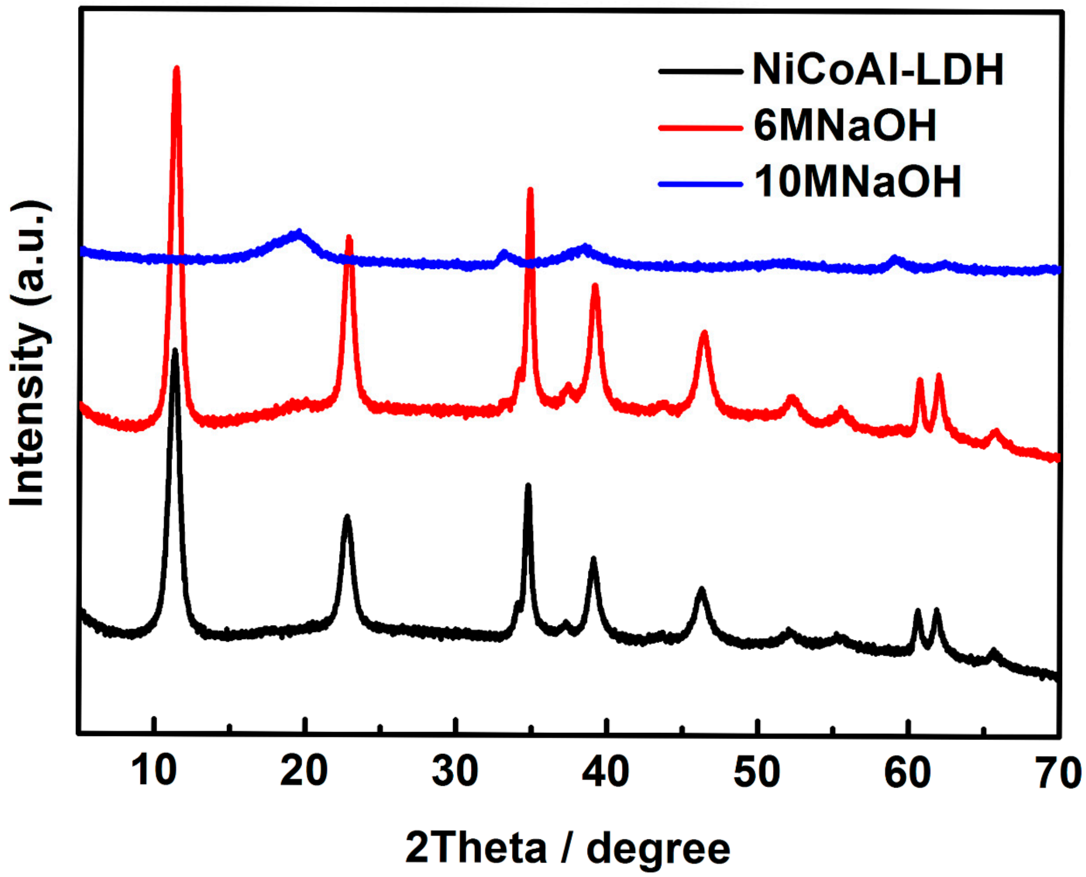

Figure S2. XRD pattern of alkali etaching NiCoAl-LDH with different concentration.
